# Characterization of aluminum composite reinforced by silver nanoparticles

**DOI:** 10.1038/s41598-023-45059-6

**Published:** 2023-10-20

**Authors:** H. Gassour, Gamal El-Din Ali Abu El-Magd, Asaad Mazen, Ahmed Mohamed Mahmoud Ibrahim

**Affiliations:** 1 Mechanical Engineering Department, Higher Technological Institute at 10th of Ramadan City, Ramadan, 44629 Egypt; 2 Production Engineering and Mechanical Design Department, Faculty of Engineering, Minia University, Minya, 61519 Egypt

**Keywords:** Materials science, Engineering, Mechanical engineering

## Abstract

This research investigates the mechanical properties improvement of reinforced Al6061 using powder metallurgy (PM) route. It utilizes the nanoparticles strengthening mechanism to enhance the prepared samples’ mechanical properties. For this purpose, Silver Nano-Particles (AgNPs) with contents of 0 wt%, 1 wt%, and 2 wt% were used to reinforce the Al6061 Matrix Composition (AMC). For optimization purposes, many parameters have been considered during the research journey namely: compaction pressure, sintering temperature, dwelling time, and reinforcement contents. All samples are examined in terms of density, hardness, and compression strength. Additionally, scanning electron microscopy (SEM), energy-dispersive X-ray (EDX) spectroscopy, mapping analysis, and XRD were used to investigate the microstructure. The experimental results revealed highest hardness values at 2 wt% AgNPs reinforced sample that was sintered at 600 °C, while the lowest was obtained at un-reinforced sample sintered at 550 °C. Furthermore, the compression strength of sample reinforced with 2 wt% AgNPs and sintered at 600 °C recorded an improvement of 25.8% of the maximum compression strength compared to the un-reinforced sample, as it reached 216 N/mm^2^. Regarding microstructural analysis, SEM imaging and EDX played an important role in exploring and interpreting many unusual test behaviors including the formation of intermetallic bonding, minor pores formation, and reinforcement dispersion un-homogeneity.

## Introduction

Excellent characteristics of Metal Matrix Composition (MMC), such as higher strengths, lower densities, and improved modulus of elasticity, make them pioneers amongst other techniques^1^. A variety of matrix materials, such as Cu, Mg, and Al, are used in MMCs nowadays^2^. Aluminum alloys 6^th^ series have good machinability that qualifies them for many industrial applications^3^. Mechanical properties can be enhanced using nano dispersion routes^4^. Particles of nano-Ag are gaining researchers' attention as reinforcement to aluminum matrix composites (AMC). While the strengthening mechanism responsible for the improvement of mechanical properties has been literately reviewed and the potential of this new group of materials was carefully studied^5^. Higher mechanical resistance of AMCs is the result of several strengthening mechanism contributions, namely (a) Load transfer, (b) Hall–Petch, (c) Orowan, (d) Coefficient of thermal expansion, and (e) Elastic modulus mismatch. The contributions sum can be calculated using the formula, while each strengthening mechanism contribution can be determined using some unique mathematical relations^5^. Different techniques such as PM and casting can be used in MMC materials production^6^. When we compare the casting technique to powder metallurgy (PM) technique, the advantages of PM outperform. In PM production, no chemical reactions occur since fabrication occurs at temperatures lower than melting point^7,8^.

1 $${\sigma }_{C}={\sigma }_{m}+\sqrt{{\sum }_{i}^{\infty }\left(\Delta {\sigma }_{i}^{2}\right)}$$

where σ_*C*_ final composite strength, Δ*σ*_*i*_ single strengthening effects, σ_*m*_ original yield strength of the unreinforced matrix.

Nano-silver was used intensely to improve some physical and medical properties of some aluminum alloys. Such improvements helped to explore new characteristics and applications for these alloys^9^. Carbon-coated silver nanoparticles proved to vastly enhance the mechanical properties of Al2024. Such behavior is believed to be due to the micro-structural nano behavior resulting from dispersing nanoparticles into Al2024 matrix. Further studies have been done to investigate the effect of the nano-silver on microstructure, and mechanical and tribological properties of cast Al6061 alloy with different amounts of nano-silver produced by the stir-casting method. Produced samples were characterized by hardness, tensile, compression, and wear tests^10^. The study results revealed that the magnitude of hardness increased evidently with the increase of the mass fraction of the nano-Ag particles. There is also an increase in compressive strength, ultimate tensile strength, elongation, and wear resistance of the Al–Ag composites compared to base alloy characteristics. Moreover, as Al-6061 alloy offers the promise of a low cost and high strength-to-weight ratio that meets many production sector demands of powder metallurgy, investigating sintering parameters was of great importance^11^. For that purpose, the investigated parameters were: die compaction pressure, sintering temperature, holding time, and heating rate. The optimum conditions were established using the Taguchi method. The analysis showed that the optimum sintering conditions corresponded to the highest compaction pressure, lowest sintering temperature, slowest heating rate, and shortest holding time. For that reason, some sintering parameters were considered while preparing test samples for extreme investigation.

In 2022, Arshad and others investigated the effect of using silver nanoparticles against multidrug-resistant microorganisms (MDR). They addressed many challenging public health issues focusing on scientific community contributions. They produced silver nanoparticles using Aloe Vera extract (Av-AgNPs) and the resultant conjugate exhibited remarkable potential to limit MDR pathogens growth^12^. Nano synthesis was followed by detailed characterization using scanning electron microscopy (SEM), X-ray diffraction (XRD) and Inductively Coupled Plasma Optical Emission Spectroscopy (ICP-OES). The so-characterized NPs showed growth inhibitory effects on multiple microorganisms.

A reduction in the counts of E. coli units (present in contaminated drinking water) was also observed when the filter paper was enveloped with Av-AgNPs. From that perspective, AgNPs contributions to healthcare sector have motivated us to investigate its implementation challenges when dispersed in AMC.

This work aims to investigate and evaluate strengthening mechanisms results offered by dispersion of AgNPs into AMC using mechanical dispersion and powder metallurgy route. The produced new composite characteristics gained anti-bacterial resistance as well as improved mechanical properties by such dispersion owing to Orowan strengthening mechanism. In this study, non-reinforced Al6061, and nano reinforced Al6061 composite materials were produced using the conventional process of PM at different sintering temperatures. The microstructure investigations were done using scanning electron microscopy (SEM), X-Ray Diffraction (XRD), Energy-dispersive X-ray spectroscopy (EDX), and Mapping analysis. Meanwhile, the mechanical properties (hardness, and compression strength) were investigated.

## Experimental procedures

For intact preparation of investigation specimens, Al6061 powder was mixed with predetermined amounts of AgNPs. For coherent samples, compaction pressure and sintering conditions formed a remarkable challenge that was surmounted by proper optimization procedures.

The powder metallurgical route was followed, and procedures were repeated several times for optimization and statistical compliance, hence, the preparation steps were mentioned abruptly. It is worth mentioning that many samples were destroyed and repeated while trying to conform to approved testing standards at many investigation stages due to mismatched sampling conditions.

### Materials and methods

The Al6061 powder used was commercially brought with an average particle size of 20–30 μm. The powder was characterized by an X-ray diffractometer (XRD), while the chemical composition was obtained as shown in Table 1 (tests were done at Science and Technology Center of Excellence-STCE-Egypt). The reinforcement particles (AgNPs) were purchased from Science and Technology Center of Excellence-STCE laboratories, then it was characterized by XRD and scanning electron microscopy SEM at Central Metallurgical Research and Development Institute (CMRDI—Egypt) and found to have average size of 30–90 nm^13^. Moreover, the average particle sizes were experimentally calculated using full width at half maximum (FWHM) of XRD peaks as per Scherer’s Eq. (2)^14,15^.

2 $$ {\text{D}}_{{\text{p}}} = \, \left( {{\text{k}} \cdot \lambda } \right)/\left( {{\text{B}} \cdot {\text{cos }}\theta } \right) $$

where, D_p_ = average crystallite size, λ = X-Ray wavelength, B = FWHM of the diffraction peak, θ = Diffraction angle, and K = Scherer’s constant.

**Table 1:** Chemical composition of Al6061 powder.

Chemical element	Al	Si	Mg	Fe	Cu	others
Composition %	97.7	0.6	1	0.4	0.25	0.5

The properties of the used powders (AMC and AgNPs) and samples’ mixing ratios were listed in Table 2.

**Table 2:** Properties of used materials in the study.

Material/property	Al6061%	AgNPs %	Theoretical density (g/cm^3^)	Melting temp (°C)
Ag nano powder	0	100	10.49	620^16^
Ag bulk	0	100	10.49	961.8
Pure Al powder	100	0	2.7	670
Sample_1	100	0	2.63	670
Sample_2	99	1	2.65	670
Sample_3	98	2	2.67	670

**Figure 1:**
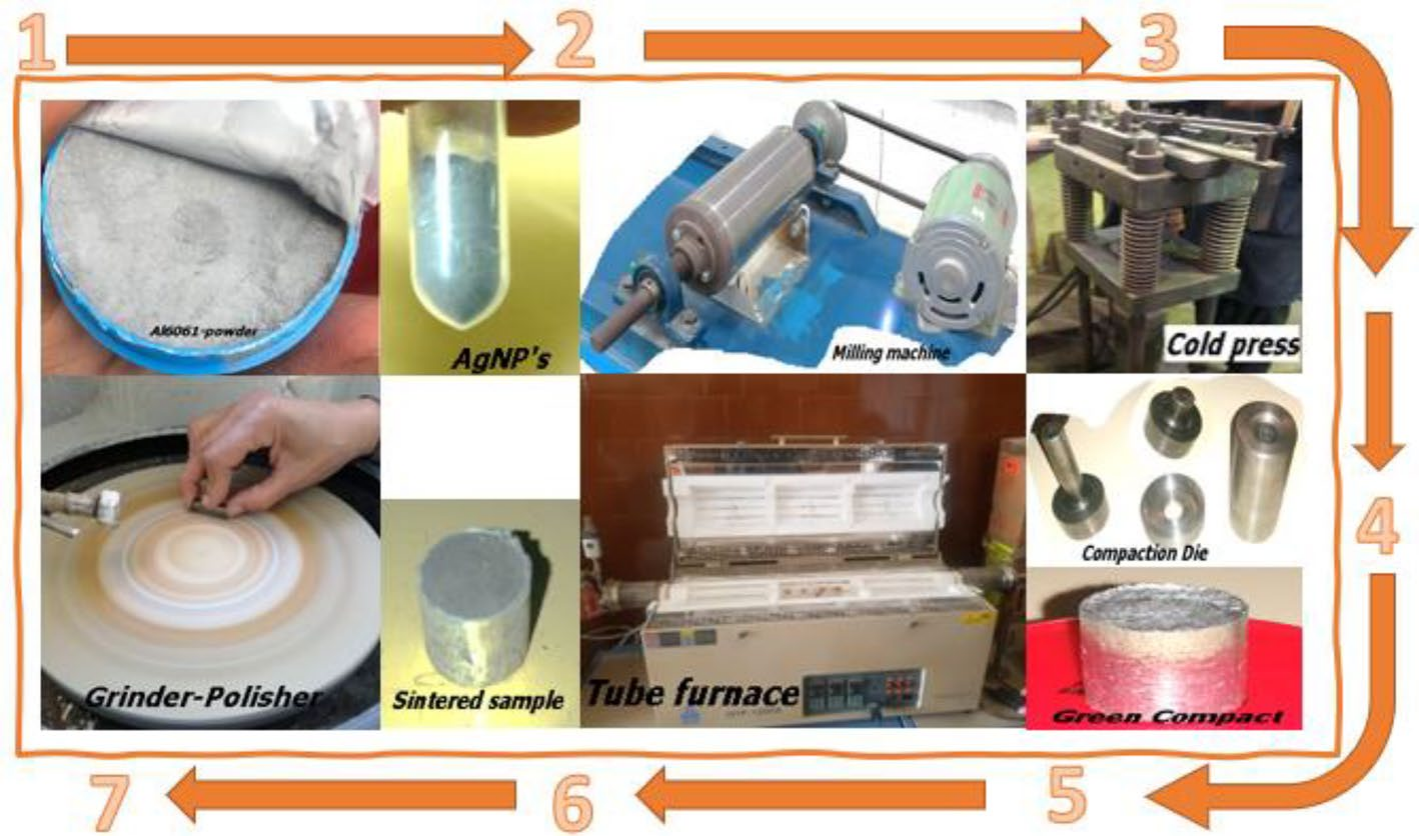
Flowchart of samples preparation.

The preparation steps started by the mixing process that was done according to predetermined ratios of the Al6061 and Ag nano powders. A milling machine was used at 150 rpm for 20 min at each mixing ratio. During mixing, the environment was kept dark as possible to limit the effects of light on the nano silver particles. Secondly, mixed amounts of powders (three amounts of 20 g each) were used to compact 12 samples at the proper compaction pressure. Each sample’s needed powder weight was calculated knowing the used die dimensions of 10 mm in diameter and the required sample height of 10 mm. Thirdly, sintering was done at two varying temperatures of 550 °C and 600 °C with constant dwelling time of 120 min. Finally, all samples were polished according to ASTM standard (MNL11187M) and prepared for the microstructural analysis phase by itching using 5 ml Hydrofluoric acid (48%), 10 ml H_2_SO_4_, 85 ml water solvent^17^. A realistic flowchart of sample preparation processes is shown in Fig. 1.

### Microstructural analysis

**Figure 2:**
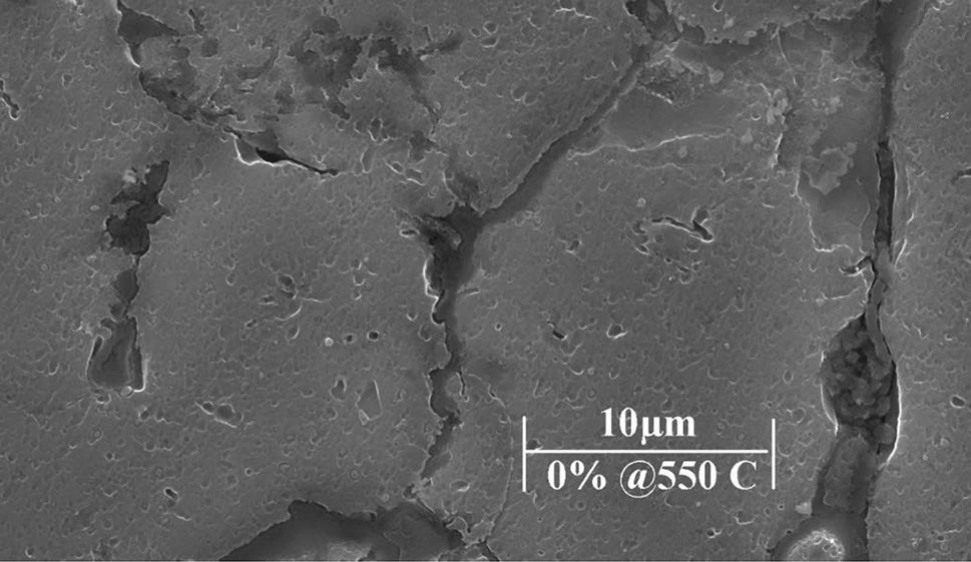
Sample SEM image of un-reinforced sample.

The used powders were tested prior to implementation for verification and declaration purposes. XRD test was performed for Al6061 and AgNPs powders, afterwards results were compared to the values of Joint Committee on Powder Diffraction Standards JCPDS cards no.89-4037 and no. 04-0783 respectively^18,19^. SEM images were also analyzed to investigate particles’ homogeneity, then compared to literatures. All prepared samples were tested using SEM (model- FEI Inspect S50), Fig. 2 shows a sample SEM image with magnification 10,000 X that is capable of clarifying samples’ grains shape and boundaries. The dispersion of AgNPs inside the AMC samples will be evidently discussed. The AMC powders had almost spherical forms with an average grain size of 30–60 μm as indicated by merchant data sheet, while the reinforcement particles’ sizes ranged from 30 to 90 nm as confirmed by calculations and SEM images with spherical shape.

For the investigation, six samples were prepared at each sintering condition. At the first condition, three mixing ratios were prepared (two samples of each mixing ratio), while the same was done at the second sintering condition. Energy-dispersive X-ray spectroscopy (EDX) and Mapping analysis were additionally performed on the same SEM images as well as other critical spots for further perception.

### Density analysis

Density measurement was made, prior to mechanical properties testing, by measuring the dry and wet weights of each specimen using a sensitive balance (0.0001 g sensitivity), then applying Archimedes method to acquire theoretical and experimental densities in order to calculate the Densification ratios for further investigations.

### Hardness and compression strength analysis

The Vicker’s method was used to investigate the hardness of the prepared samples produced by the indicated PM route. Measurements were made according to ASTM E384 using a Digital Micro Vickers Hardness Tester, (Model: Bht 1000) loaded with 1KgF and a dwelling time of 13 s. Additionally, only three produced samples were selected for mechanical compression test using a universal machine (Model: UTM-1000KN). Nevertheless, general requirements of testing and calibration laboratories (ISO/IEC 17025) were considered while performing all test procedures for boosted results reliability and improved data confidence level.

## Experimental results

### Microstructure and density characterization

**Figure 3:**
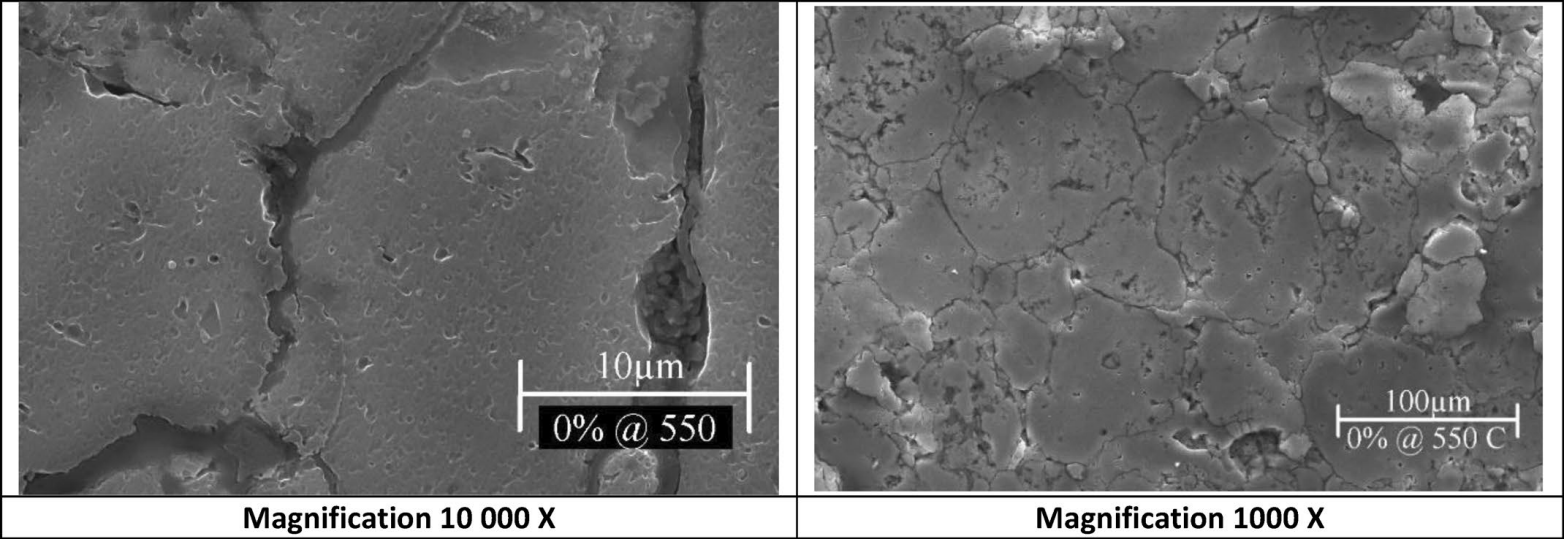
SEM images of 0 wt% Nano silver sample sintered at 550 °C at different magnifications.

**Figure 4:**
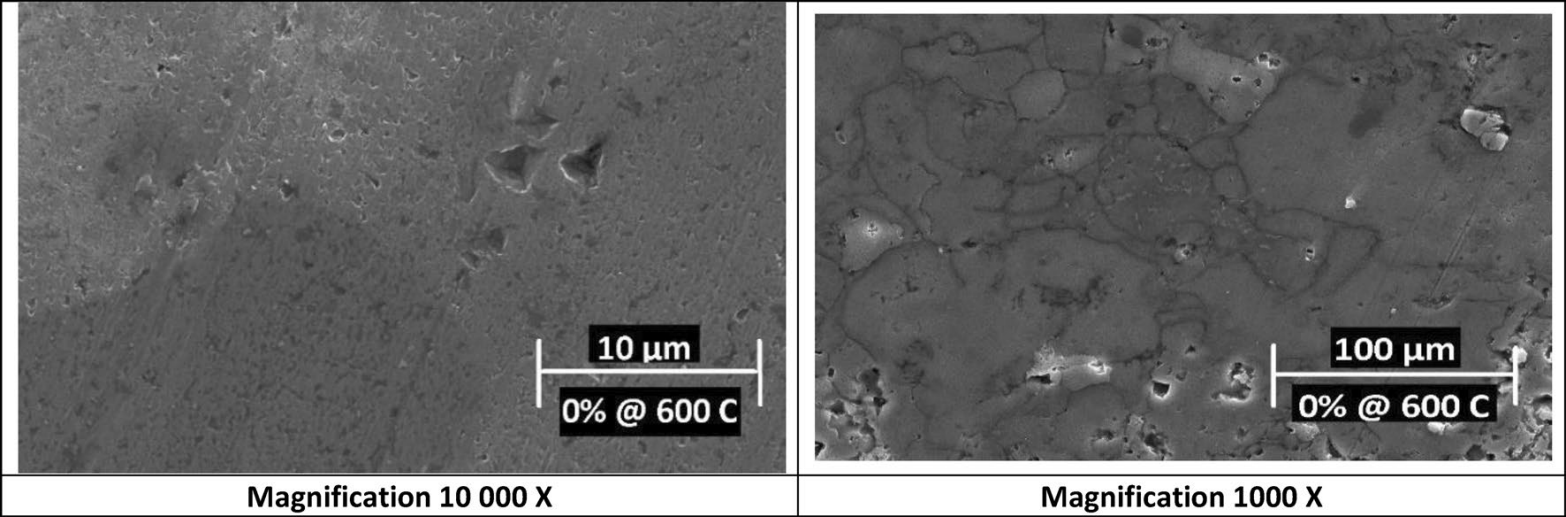
SEM images of 0 wt% Nano silver sample sintered at 600 °C at different magnifications.

As stated before, SEM was used to characterize the produced samples by showing microstructures and particles distribution at each sampling condition. For improvements detection evidence, SEM images of the un-reinforced Al6061 samples sintered at 550 °C and 600 °C were obtained in Fig. 3 and Fig. 4 respectively. It demonstrates no major defects in the microstructure, such microstructural behavior resulted from the high density of AMC that occurred by proper compaction pressures and adequate sintering conditions.

Furthermore, samples sintered at 550 °C showed smaller average grains sizes than samples sintered at 600 °C, which could be attributed to the fact that higher sintering temperatures give much energy to the grain’s boundary that may causes grain growth^20^.

**Figure 5:**
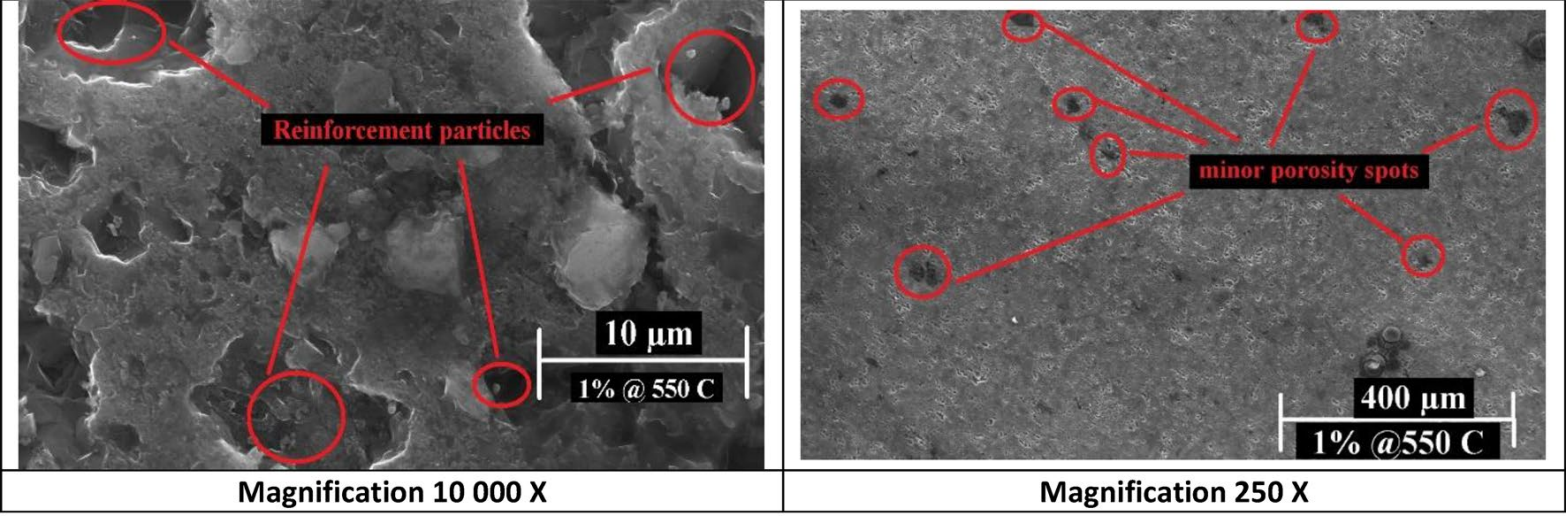
SEM images of 1 wt% Nano silver sample sintered at 550 °C at different magnifications.

**Figure 6:**
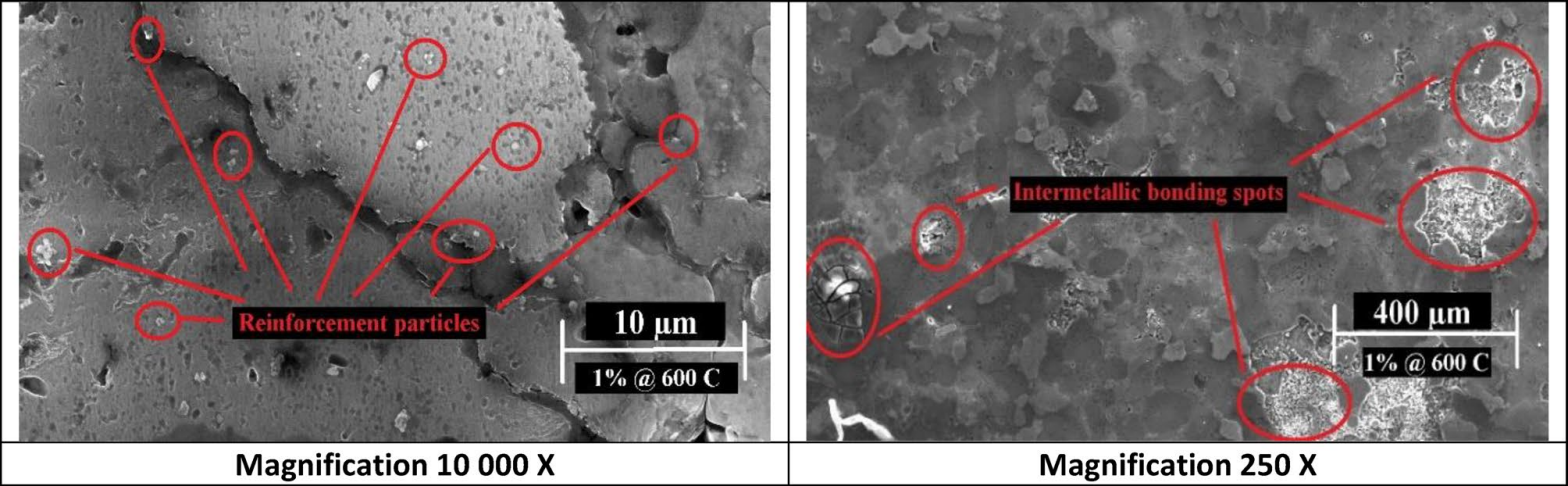
SEM images of 1 wt% Nano silver sample sintered at 600 °C at different magnifications.

The SEM images of reinforced samples’ microstructures mostly demonstrated homogenous matrix dispersion. Samples with 1 wt% nano silver content sintered at 550 °C and 600 °C (Figs. 5 and 6 respectively) showed minor porosities at some captured spots.

**Figure 7:**
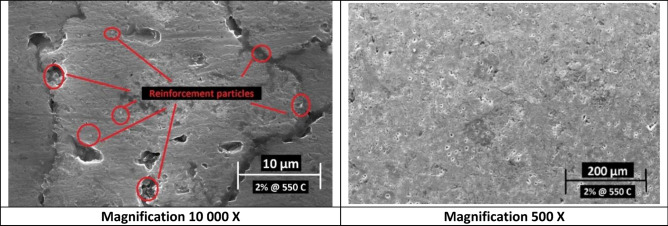
SEM images of 2 wt% Nano silver sample sintered at 550 °C at different magnifications.

**Figure 8:**
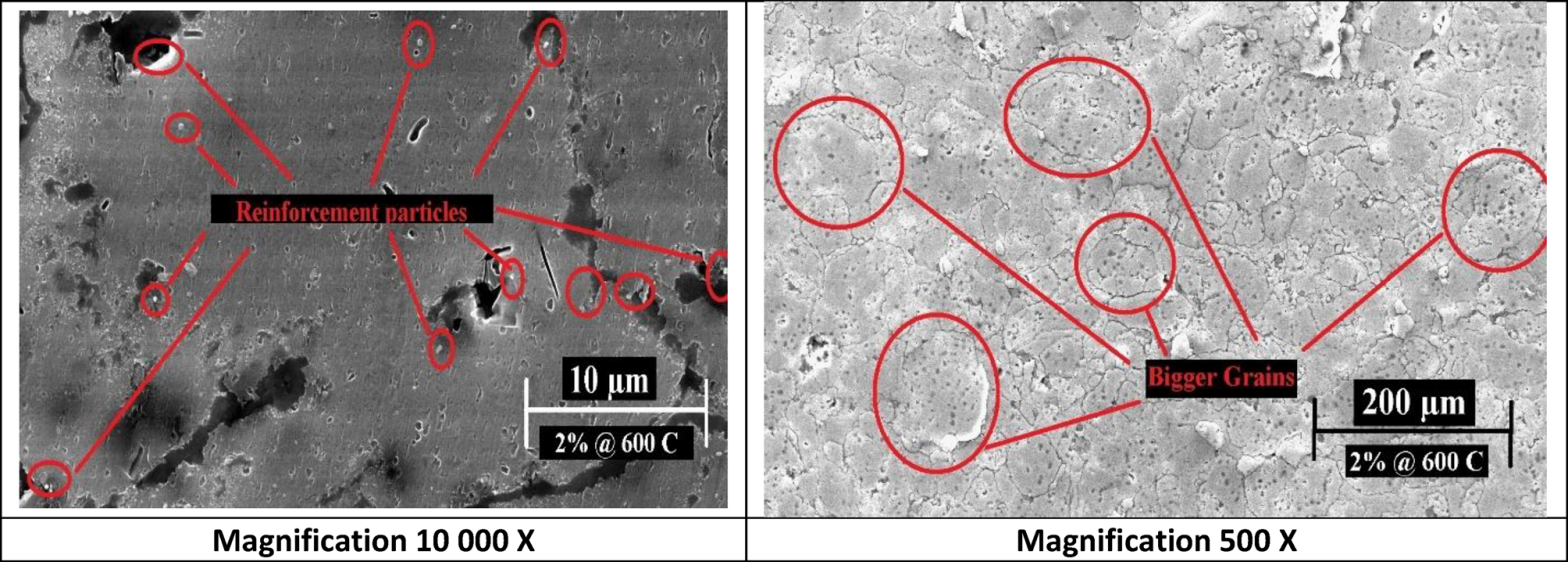
SEM images of 2 wt% Nano silver sample sintered at 600 °C at different magnifications.

However, samples having 2 wt% Nano silver contents showed homogenous distribution and minor agglomerations at indicated spots that were characterized and confirmed later by EDX and mapping analysis. Figures 7 and 8 show the SEM images for these samples that were sintered at 550 °C and 600 °C respectively.

Generally, the number of pores formed in the sample’s microstructure were adequate and within the acceptable limits of PM sampling standards^21^. Moreover, little micro cracks were observed in samples.

Such microcracking behavior is consistent with literature that focused on E-SEM micro graphing of tapered die compacts with relative densities of 95%. Such compacts were subjected to exhibit diffuse surface microcracking^22^. From the past figures, one can conclude that sintering at 600 °C led to larger grains sizes compared to sintering at 550 °C. This change in microstructure, grains sizes, would evidently affect mechanical properties and reinforcement percentages as well.

**Figure 9:**
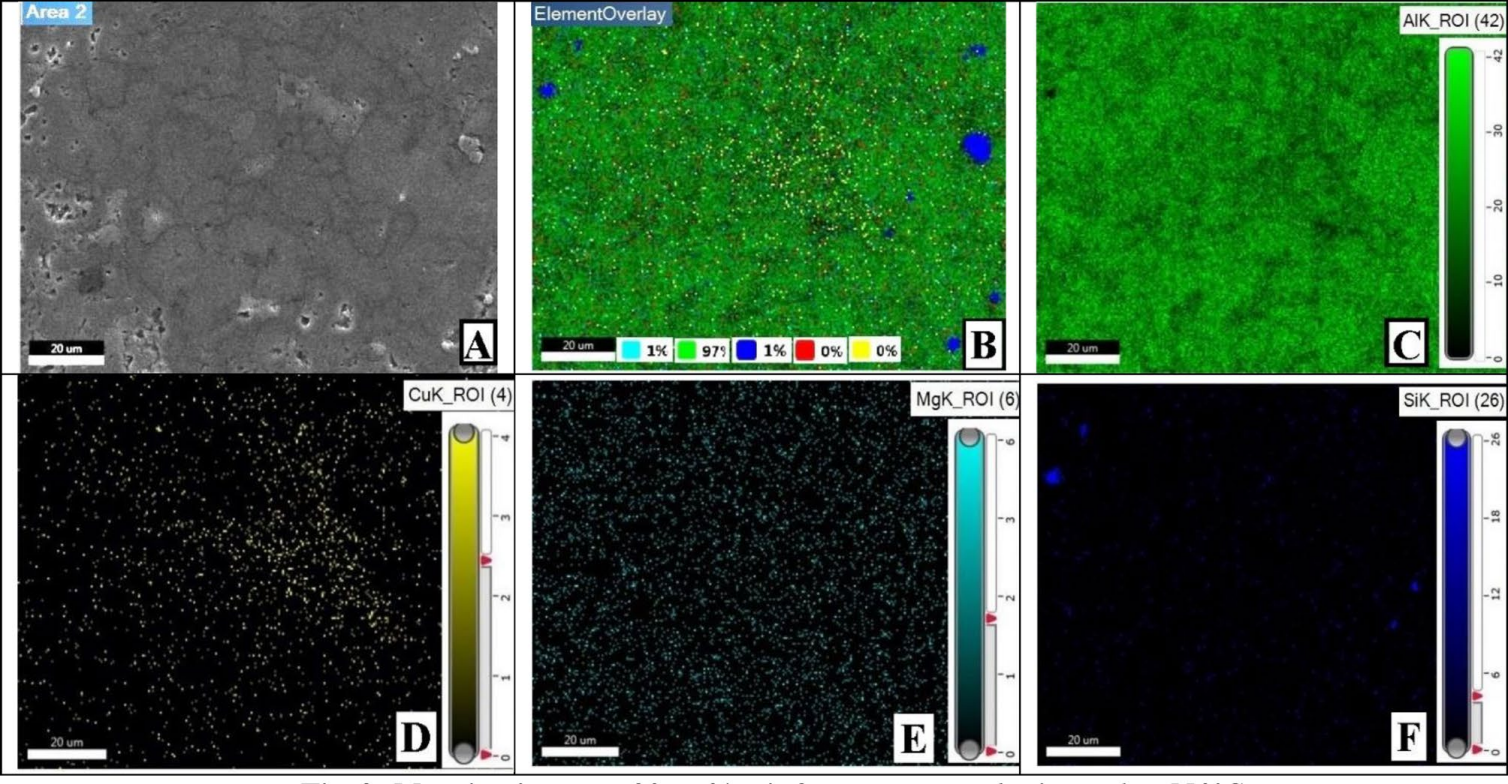
Mapping images of 0 wt% reinforcement sample sintered at 550 °C.

As this study focuses on AMC reinforcement, small amounts of Mg, Si, Fe, and Cu were out of the analysis scope, which is why they are not extremely considered in mapping imaging analysis. Mapping images and EDX analyses for the prepared samples were presented in order to display and assure particles distribution uniformity. Therefore, four samples’ analyses will be displayed and discussed in order to clarify different investigation parameters ‘potential effects. The samples mapping of (0 wt% AgNPs at 550 °C), (0 wt% AgNPs at 600 °C), (2 wt% AgNPs at 550 °C), and (2wt% AgNPs at 600 °C) correspond to Figs. 9, 12, 14, and 16, respectively.

**Figure 10:**
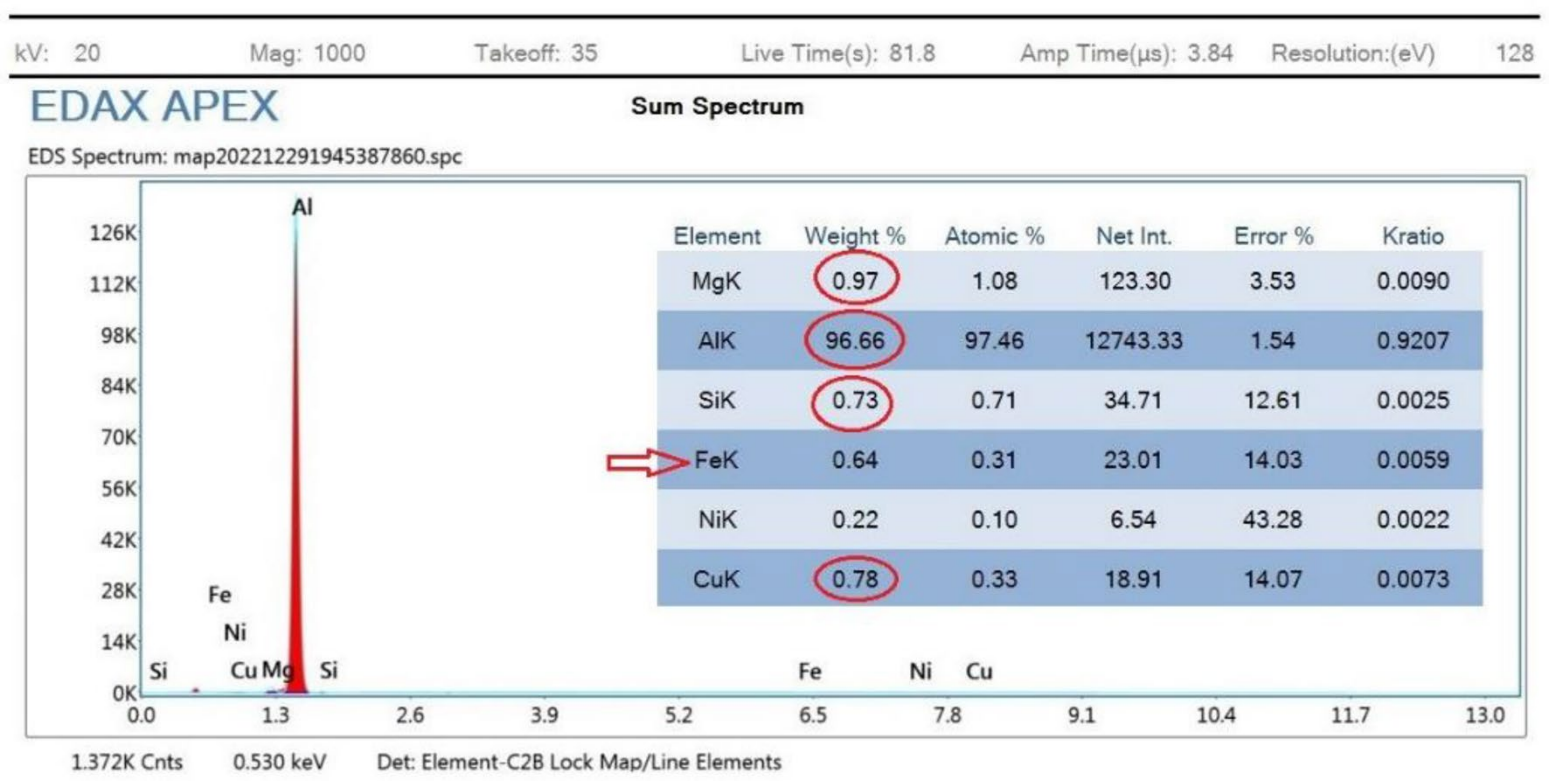
Sample mapping spectrum analysis with indicated constituents’ percentages.

For that reason, Fig. 9 conforms the uniformity of particles distribution of un-reinforced sample sintered at 550 °C at one selected spot. At (Fig. 9B) the overlay image indicates minor agglomerations spots that have been confirmed to be Si particles. It could be attributed to inadequate mixing or sintering conditions. The spectrum presented in Fig. 10 conforms to the indicated mapping inference; it shows the precise constituents’ percentages. It could be remarked that a small amount of Fe is present in this sample spot. Such a small percentage could be attributed to milling process effects due to the metallic balls friction loss while mixing.

**Figure 11:**
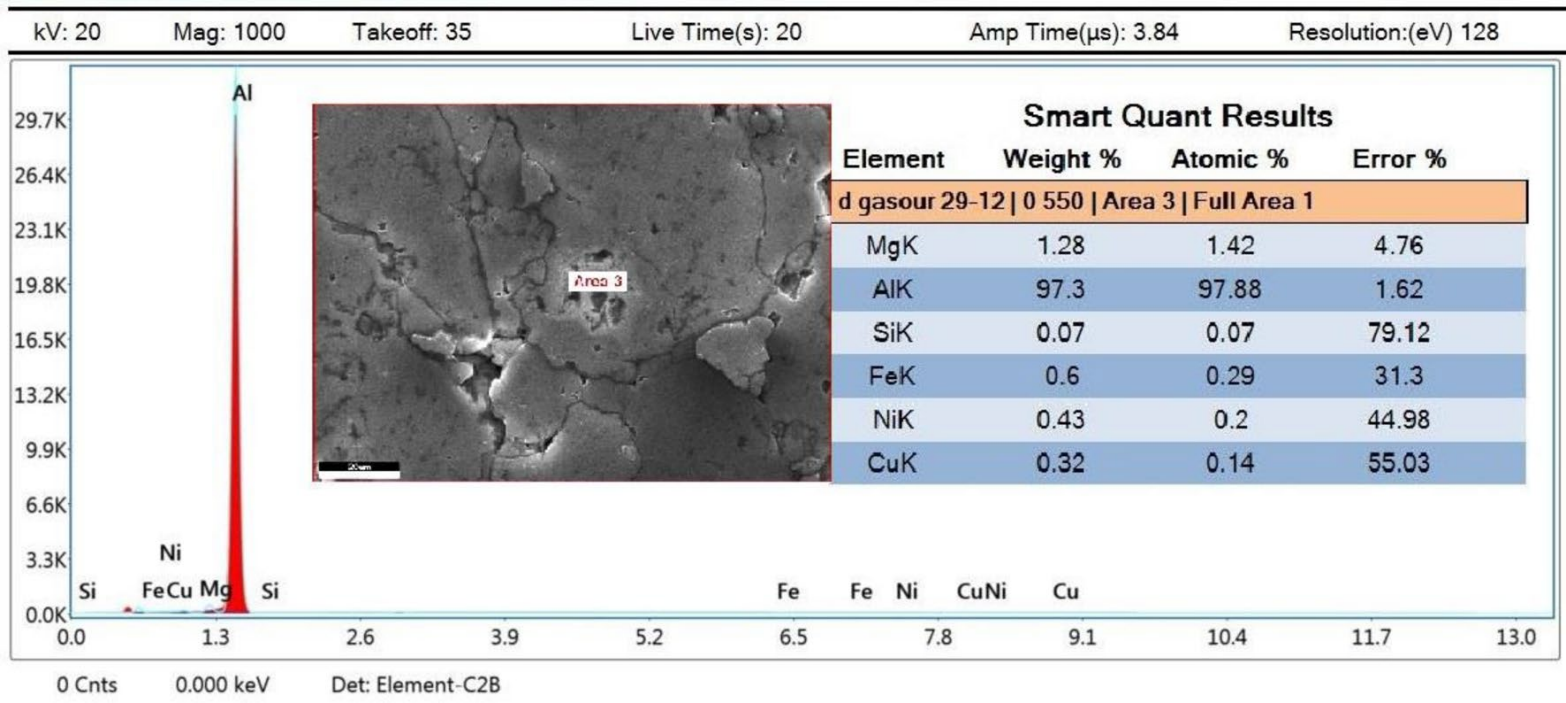
EDX analysis for 0 wt% reinforcement sample sintered at 550 °C.

For further constitutions verifications, EDX analyses were used at several spots to positively contribute to characterization process. Figure 11 illustrates the analysis spot for a selected area for un-reinforced sample sintered at 550 °C.

**Figure 12:**
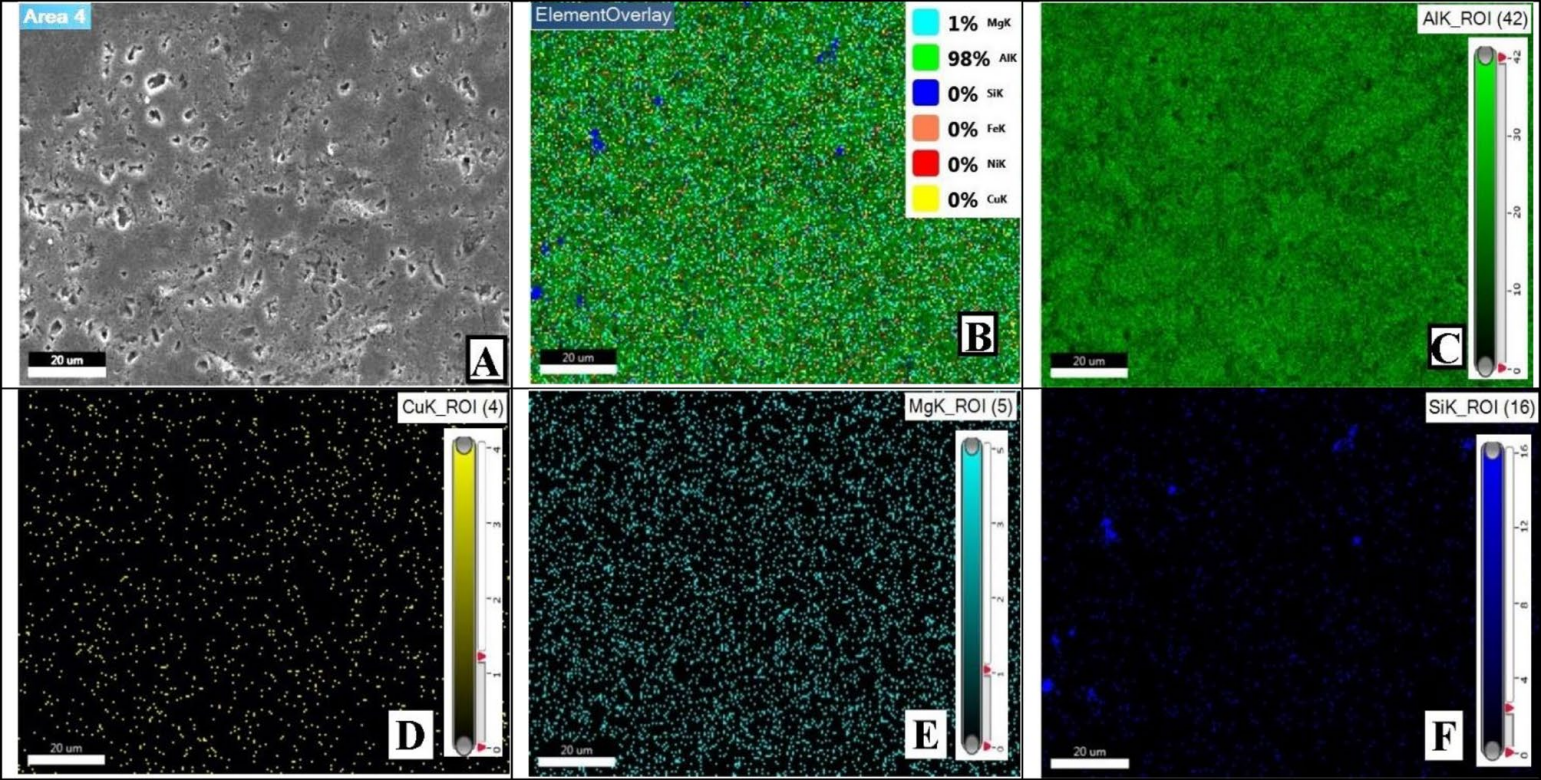
Mapping images of 0 wt% reinforcement sample sintered at 600 °C.

For optimization purposes, SEM imaging, EDX, and Mapping analysis were repeated for all samples at all sampling conditions and at many spots in each sample. Consequently, Fig. 12 shows mapping analysis for the un-reinforced sample sintered at 600 °C in order to clarify the sintering condition changing effects.

**Figure 13:**
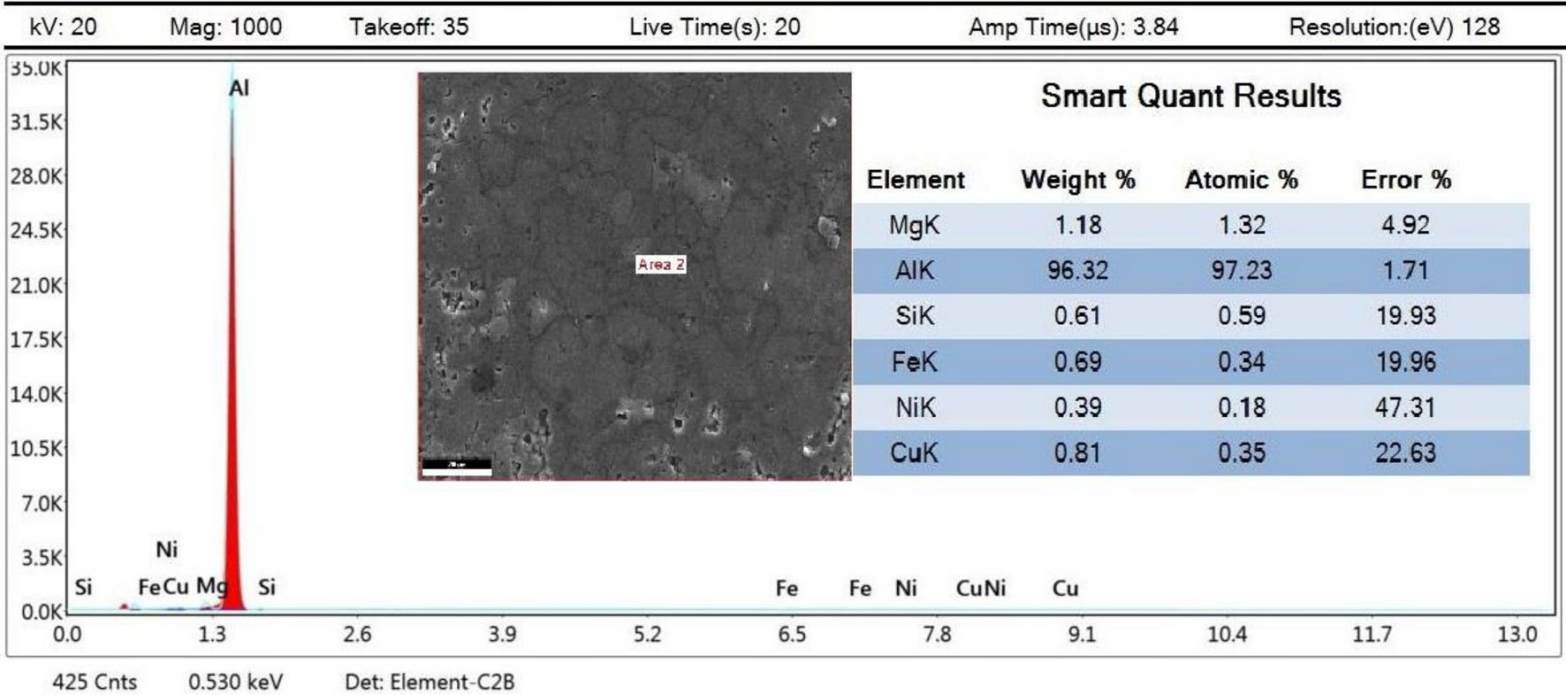
EDX analysis for 0 wt% reinforcement sample sintered at 600 °C.

It is obvious by comparing mapping analyses of the samples having the same composition (Figs. 9, 12) but sintered at two different temperatures that higher sintering temperature attained better particles distribution and dispersion of all constituents. Figure 13 shows the EDX analysis for unreinforced sample sintered at 600 °C. It showed no major changes regarding particles distribution and composition.

**Figure 14:**
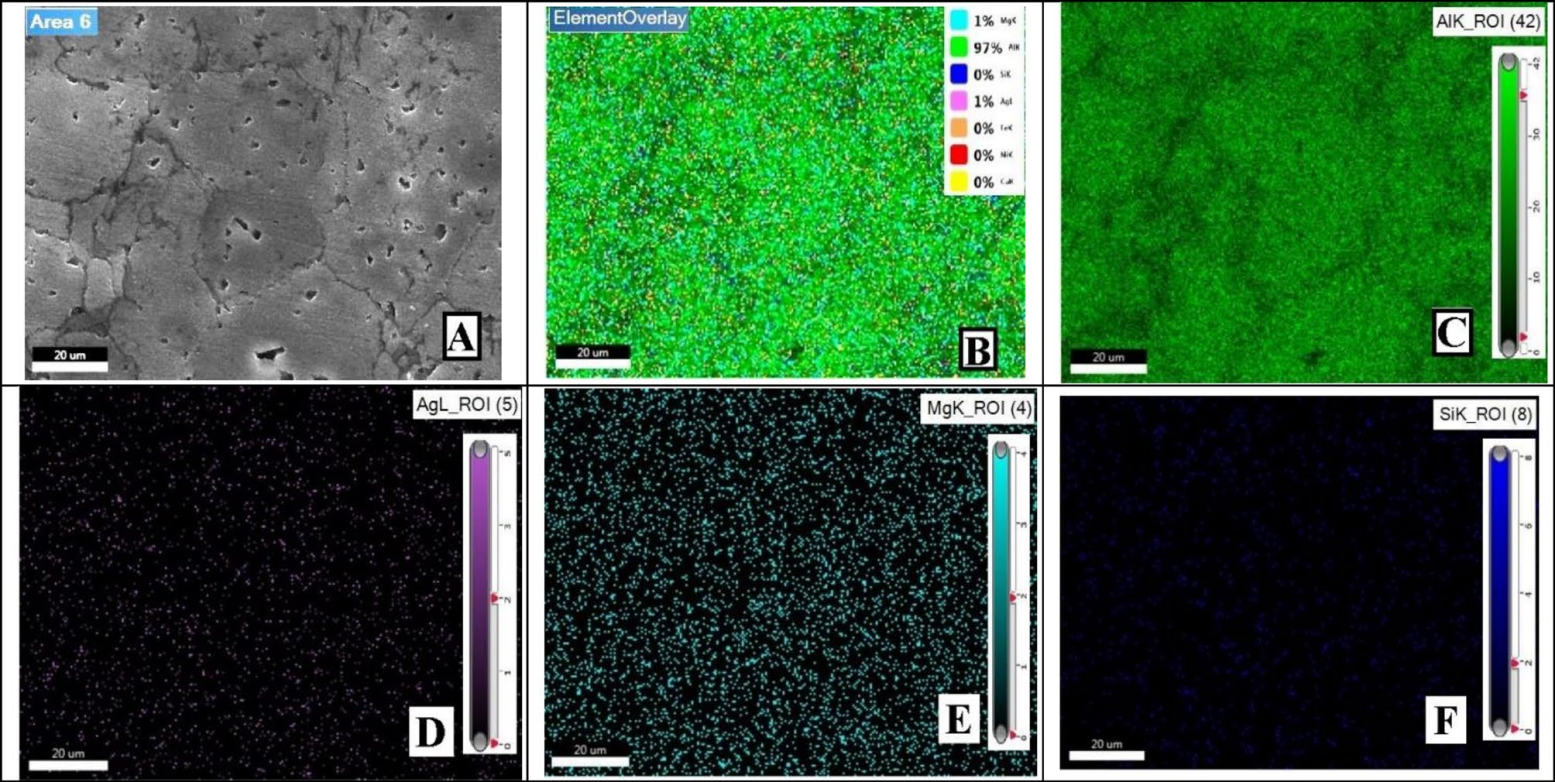
Mapping images of 2 wt% reinforcement sample sintered at 550 °C.

Mapping analysis, at selected area, of the sample with 2 wt%. reinforcement content sintered at 550 °C is presented in Fig. 14. It is evident from Fig. 14D that reinforcement particles (AgNPs) were properly dispersed through AMC.

**Figure 15:**
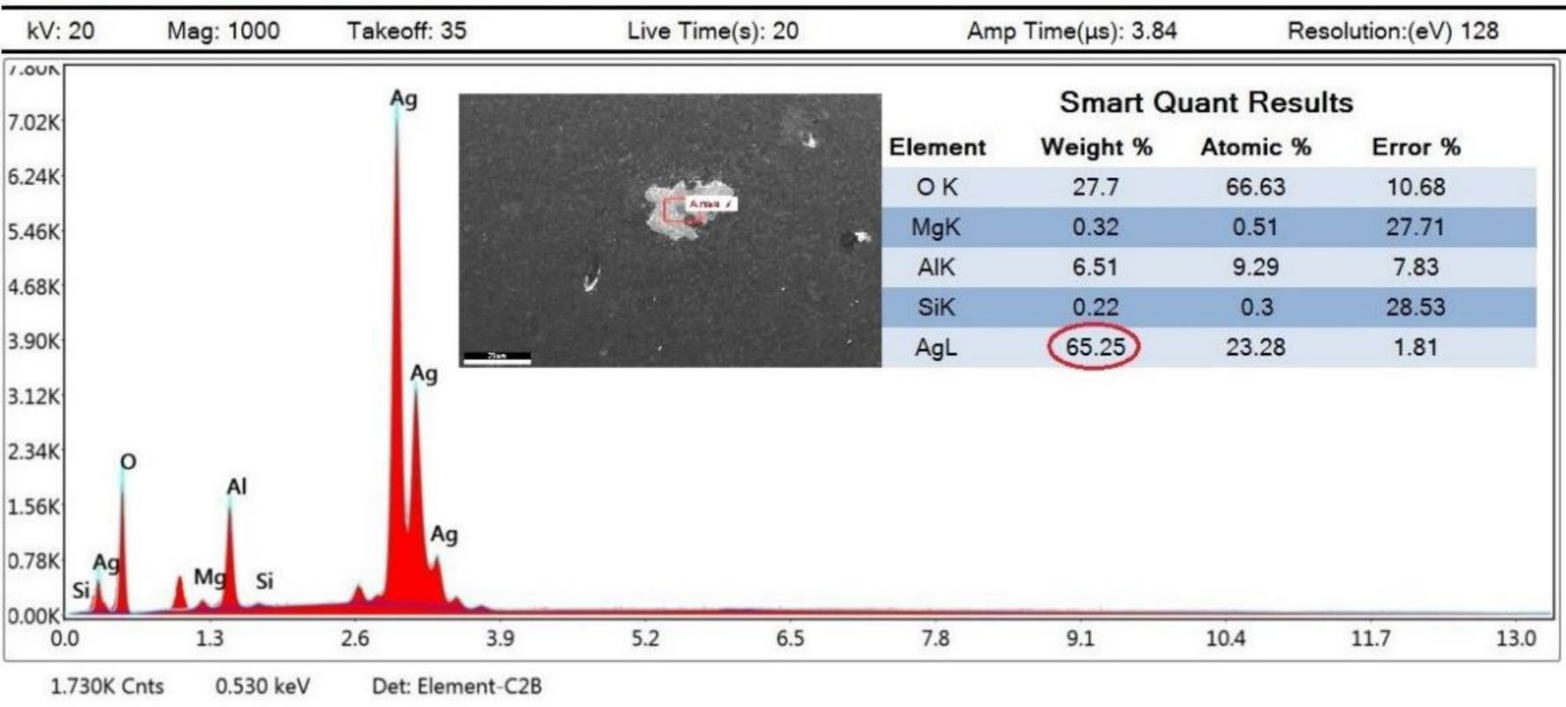
EDX of defected spot analysis for 2 wt% reinforcement sample sintered at 550 °C.

The corresponding EDX analysis for the same sample spot showed no abnormality, but another spot presented in Fig. 15 showed moderate AgNPs agglomerations. Such defect slightly affected the investigated mechanical properties as will be shown later.

**Figure 16:**
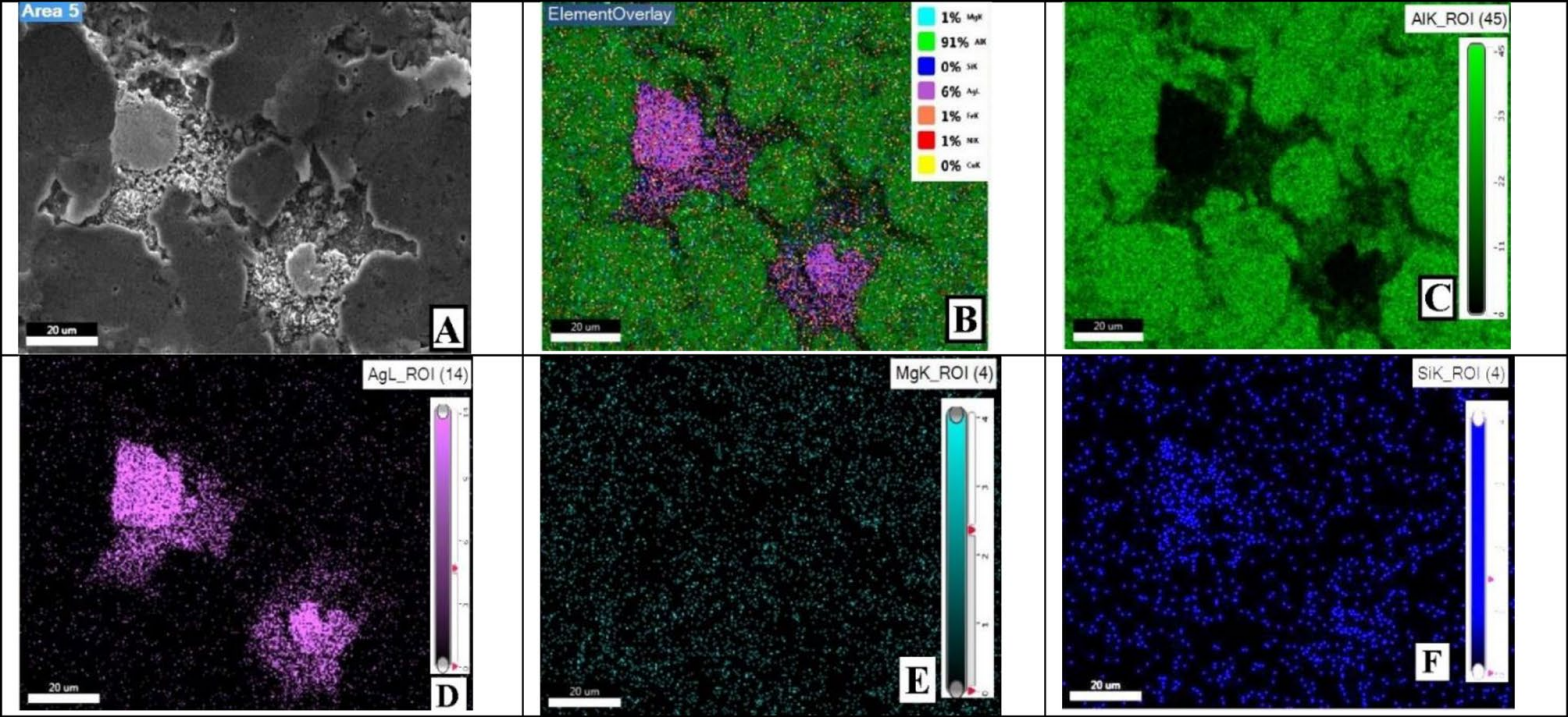
Mapping images at defected spot of 2 wt% reinforcement sample sintered at 600 °C.

Moreover, mapping analysis, at selected areas, of the sample with 2wt%. reinforcement content sintered at 600 °C showed normal behavior. Unless for one spot presented in Fig. 16. It is evident from Fig. 16D that reinforcement particles of AgNPs agglomerated at this point. It could be attributed to inadequate mixing process. Such revealed defects may possibly imply the existence of other unrevealed ones. Nonetheless, minor defects were analyzed and thoroughly addressed later in terms of mechanical properties effects.

**Figure 17:**
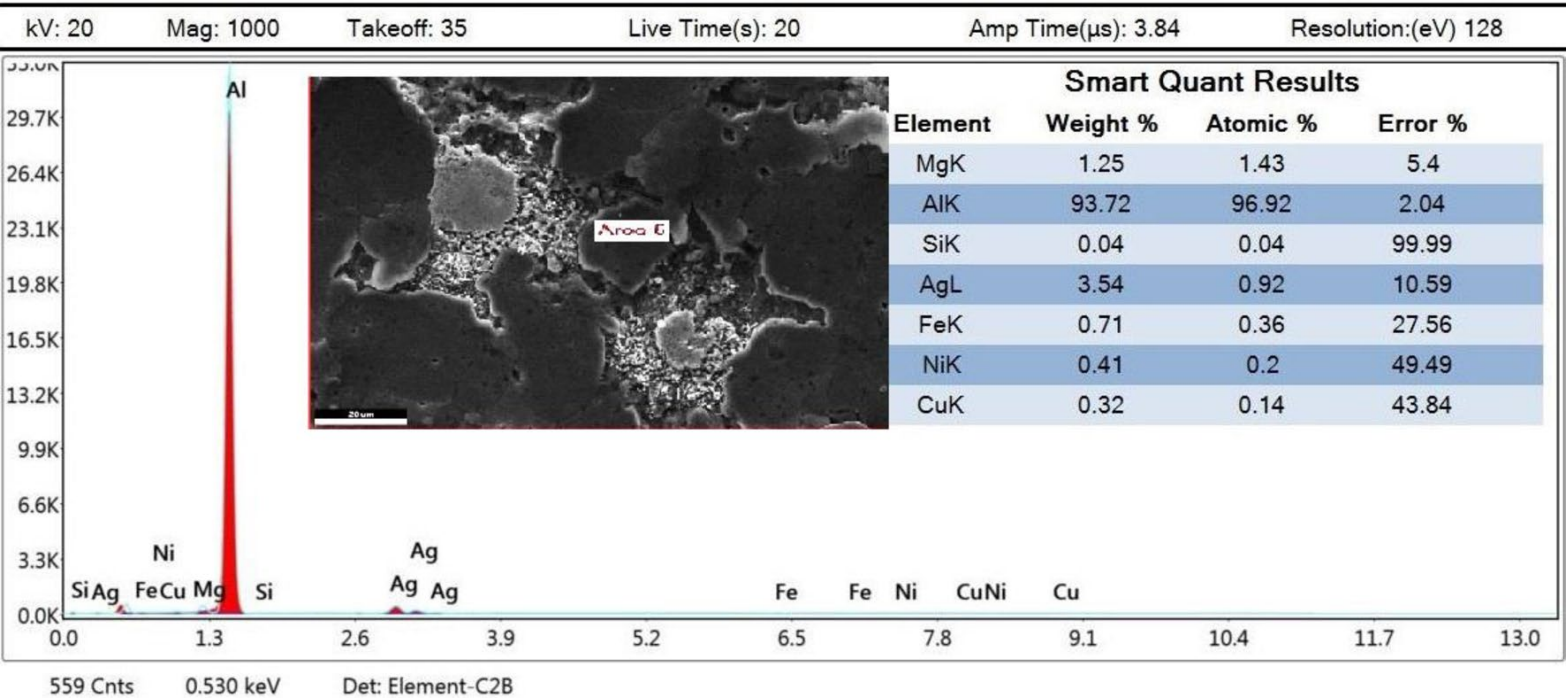
EDX of defected spot analysis for 2 wt% reinforcement sample sintered at 600 °C.

**Figure 18:**
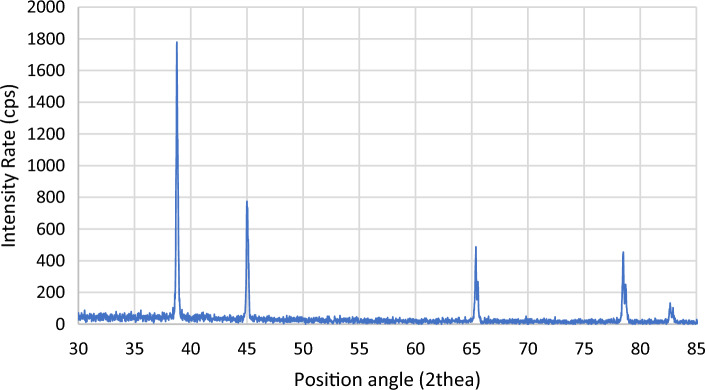
XRD Al6061 powder.

**Figure 19:**
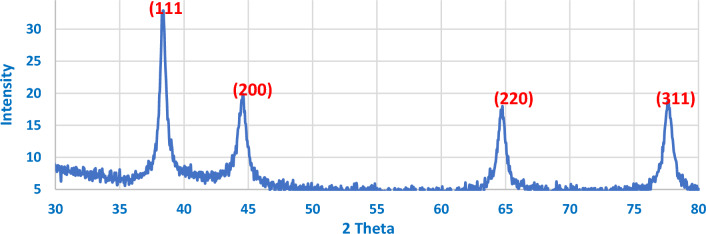
XRD graph of AgNPs showing four characteristic peaks.

The EDX analysis at the indicated area shown in Fig. 17 shows the precise composition of this sample spot having 2 wt% reinforcement contents and sintered at 600 °C. XRD peaks of Al6061 powder and Ag nanoparticles, Figs. 18, and 19 respectively, were successfully identified and compared to literature^13,23^. Such comparisons indicated that Al6061 (111) peaks are distinct and more extensive than other constituents’ peaks.

**Figure 20:**
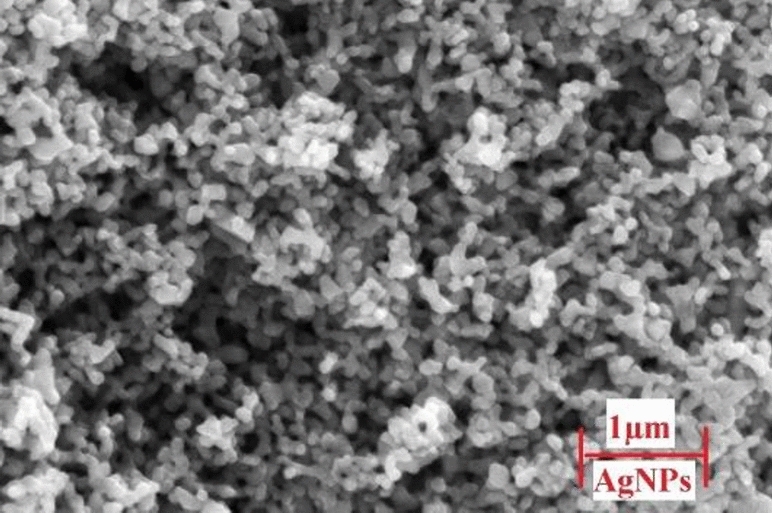
SEM image of AgNPs.

XRD analysis was also used to define the crystal structure of the used AgNPs as shown in Fig. 19. Brag reflections were noticed for 2θ values at 38.4°, 44.6°, 64.76°, and 77.6° that correspond to 111, 200, 220, and 311 lattice planes diffractions, respectively. Also, SEM image of AgNPs powder was displayed in Fig. 20.

Table 3 shows the peaks data and the corresponding FWHM values calculated using a specially coded Excel sheet. From literature, one can conclude that the AgNPs structure could be indexed as Face Centered Cubic (FCC)^24^. Peaks corresponding to 111 planes were more intense than the others. Such intensity was believed to be due to the nanosized crystal structured effect. The average NPs diameter size was calculated using Scherrer’s equation with a geometric factor of 0.89 and an x-ray wavelength of 0.15406. Correspondingly, the diameters ranged from 15.75 to 30.4 nm^25,26^. Subsequently, the highest examined strength values are expected in Al6061 samples reinforced with smaller silver particles, while lower values were expected at bigger reinforcement particles’ sizes.

**Table 3:** XRD peaks data.

	Pos (°)	Height	FWHM	Rate (CPS)
111 Peak	38.4	32.894	0.281859	1127.08
200 Peak	44.6	19.875	0.103215	411.46
220 Peak	64.76	18	0.08438	337.5
311 Peak	77.6	18.735	0.092019	365.63

This explains why some samples properties sintered at 550 °C and reinforced with higher AgNPs contents and sizes exhibited inexplicable mechanical properties compared to some other reinforced and un-reinforced samples^26^.

It is obvious from SEM and EDX that grain refinement does occur with the dispersion of AgNPs in AMC due to a reduction in grains sizes. By such dispersion and proper sintering processes, grain sizes of the main matrix decreased. Hence the strength of the samples was increased. Variant grain sizes are a key reason for distinct changes in the mechanical properties of all produced samples, as it has a direct effect on mechanical properties^27^. Consequently, mechanical properties (Hardness strength, Compression strength) of aluminum alloys are believed to be improved by proper contents dispersion of nanoparticles as reinforcements to AMC. Although Ag properties are weaker than Al, it is evidently capable of reinforcing it. It is also obvious that agglomerations increase in parallel with increasing reinforcement content.

Elementary (EDS) analyses were performed at some curious samples’ spots to illustrate and interpret mechanical properties behavioral changes. Minor wettability problems were observed between Aluminum and silver nanoparticles when used as reinforcement^28^ as have been noticed from cavities exist in some SEM images. These modest cavities had minor effects on mechanical strength that were neglected and saved for future investigation. Assuring samples intact properties, densities were measured prior to mechanical strength testing. For that reason, dry and wet weights were experimentally assigned using Archimedes’ theory, and experimental densities were attained. Also, the theoretical densities were calculated, and all data were listed in Table 4. Densification percentages indicates samples testing reliability, it is calculated as shown in Eq. (3).

**Table 4:** Theoretical and experimental densities calculations data.

Sample #	Nano %	Sintering Temp°C	Dry weight measured	Wet weight measured	Exp density (gm/cm^3^)	Theoretical density	Densification %
1	0%	600	1.8315	1.0922	2.477	2.631	94.2
2	1%	600	1.0998	0.6554	2.475	2.650	93.4
3	2%	600	1.6895	1.0085	2.481	2.670	92.9
4	0%	550	1.6417	0.9522	2.381	2.631	90.5
5	1%	550	1.2461	0.7454	2.489	2.650	93.9
6	2%	550	1.5895	0.96085	2.528	2.670	94.7

3 $$Densification=\frac{Experimental\, Density}{Theoretical\, Density}\times 100$$

**Figure 21:**
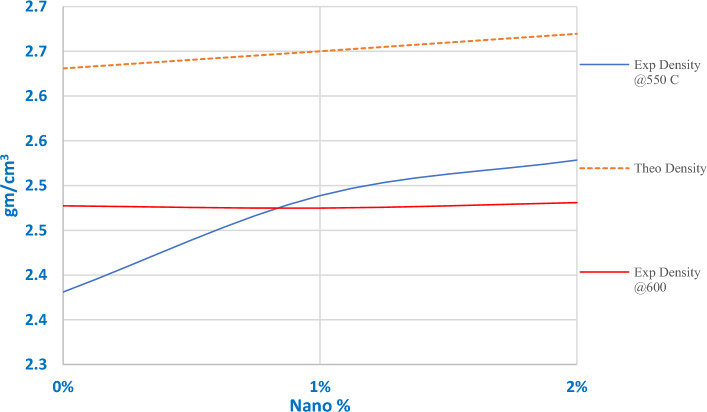
Theoretical and Experimental density relation versus AgNPs contents.

Figure 21 demonstrates theoretical and experimental density relations versus AgNPs percentages for produced samples. As Table 4 depicts, the densification factor for all samples exceeded 90% of the theoretical density^29^, such percentages should involve good mechanical characteristics. Two trends can be observed from Fig. 21, the first is for sintering at 600 °C, samples had the highest densification percentage of (94.2%) at 0 wt% addition ratio. While samples at an addition ratio of 2 wt% had the lowest density (92.9%). The second trend is for samples sintered at 550 °C; the densities increase simultaneously with increasing reinforcement ratio.

### Hardness and compression analysis

**Figure 22:**
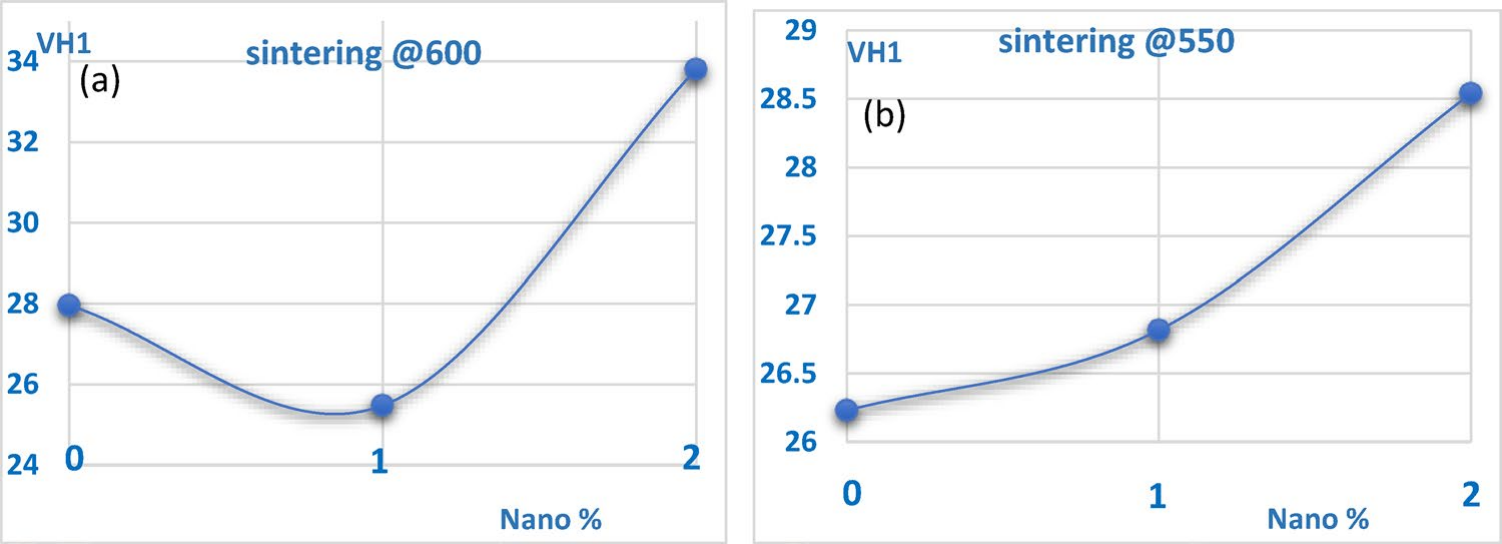
(**a**) Hardness results for samples sintered at 600 °C. (**b**) Hardness results for samples sintered at 550 °C.

Figure 22 shows the enhancement of hardness values of the produced samples. Generally, reinforced composites had higher hardness values compared to un-reinforced Al6061 samples. For instance, un-reinforced Al6061 had an average hardness of 27.97 VH and 26.23 VH for samples sintered at 600 °C and 550 °C respectively. Samples with 2 wt% addition of nano silver displayed the highest values of hardness of 33.81 VH and 28.54 VH at the same sintering conditions.

From the literature survey, hardness values of AMC reinforced with nanoparticles generally increase with increasing reinforcement contents. Therefore, test results were conforming to literature^30^. Assuring the accuracy and reliability of the test results, samples were tested three times for statistical error minimization. It is of great importance to discuss the effect of sintering temperature on the hardness values while varying the nano silver dispersion contents. Figure 14a shows the variation of hardness values while increasing nano percentages from 0 to 2 wt% when the samples were sintered at 600 °C. It is worth noting that increasing the nano silver contents in the samples by 2 wt% improved the hardness values by 20.9% at the same sintering conditions. The 1 wt% nano silver addition sample recorded unexpectedly lower hardness values; it could have been resulted from an insufficient nano addition ratio that halted the strengthening mechanisms from effectively taking place at a sintering temperature of 600 °C.

Figure 22b shows the effect of increasing nano silver content on the hardness values, and it is worth mentioning that increasing the amounts of nanoparticles in the test samples from 0 to 2 wt% increased the hardness by 8.8% at sintering conditions of 550 °C. Such improvements yielded a max hardness of 29.52 VH1 at 2% nano addition. Improvements in hardness values could be attributed to the grain refinement of AMC, which was spotted in the microstructure analysis, also the uniform dispersion of nanoparticles played a vital role in such enhancement as it acted as barriers to the motion of dislocations conferring to Orowan strengthening mechanism^27^.

Tables 5 and 6 shows the test results and the indentation marks of each trial at each sintering condition of 550 °C and 600 °C, respectively.

**Table 5:** Micro-Hardness values for samples sintered at 550 °C.

Measurement #	Nano silver (wt)	Sintering temp (°C)	Result (HV1)	Average	Indentation
1	0%	550	23.65	26.23	
2	0%	550	28.34		
3	0%	550	26.7		
4	1%	550	23.36	26.82	
5	1%	550	27.85		
6	1%	550	29.24		
7	2%	550	29.52	28.54	
8	2%	550	28.71		
9	2%	550	27.39

**Table 6:** Micro-hardness values for samples sintered at 600 °C.

Measurement #	Nano silver (wt)	Sintering temp (°C)	Result (HV1)	Average	Indentation
1	0%	600	27.75	27.96	
2	0%	600	28.12		
3	0%	600	28.01		
4	1%	600	27.15	25.48	
5	1%	600	24.72		
6	1%	600	24.56		
7	2%	600	34.70	33.81	
8	2%	600	34.31		
9	2%	600	32.43		

**Figure 23:**
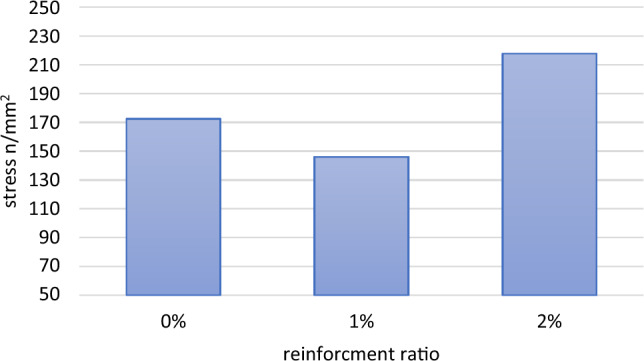
Maximum compression stress comparison.

As for further investigation of mechanical properties, the compression test was performed and showed satisfying results. Figure 23 compares the maximum endurable compression stress values for three samples having nano silver contents of 0, 1, and 2 wt%. It was observed that maximum strengths’ values improved at increased reinforcement percentages.

The sample with 2 wt% AgNPs content showed the best stress resistance values. That implies adding nano silver particles to the Al6061, as the main matrix composition, considerably improved the mechanical properties. The improvement percentages at 2 wt% addition reached 25.8% of the maximum compression stress of the un-reinforced sample.

**Figure 24:**
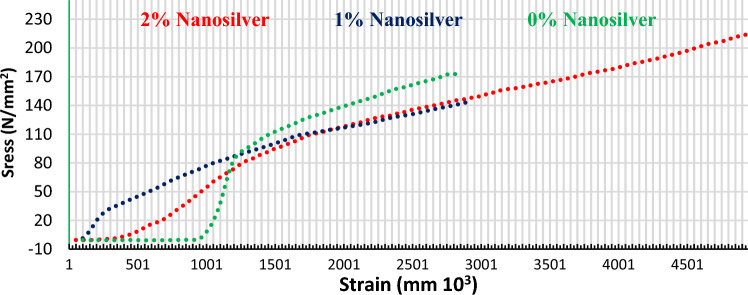
Stress–strain behavior comparison at different reinforcement contents.

Figure 24 shows the stress–strain curve behaviors for the three tested samples. The 2 wt% AgNPs contents sample showed the best behavior improvement. The enhancement in the mechanical properties in the case of the addition of the nano silver particles referred to the existence of the nanoparticles that ease the transition of the stresses which consequently decreases the possibilities of stress concentration zones^31^.

As noticed, the addition of 1% AgNPs exhibited lower than expected compression strength and hardness values at some sintering conditions, such behavior could be attributed to insufficient reinforcement addition content that was essential to support Orwan strengthening mechanisms or due to inappropriate sintering temperature of 550 °C.

Finally, AgNPs dispersed Al6061 would add-up much more value for such alloy medical-industrial applications if given more attention. As AgNPs have good proven anti-bacterial resistance and have many approved eco-friendly preparation methods compared to other reinforcements (such as Carbone coated silver nanoparticles (AgNPs-C), it is advised to address and evaluate its implementation in many critical medical applications such as artificial organs structures and surgical supplements.

## Conclusions

Al6061 was strengthened with AgNPs using the Orowan method's dispersion strengthening mechanism. This work attempted to combine the outstanding properties of aluminum with the antibacterial properties of nano-silver while mechanical strengthening is achieved. The main findings of the investigation can be withdrawn as follows:

1. The compression strength of AMC reinforced by AgNPs is impacted by the Orowan strengthening process. For instance, sintering a sample with a 2 wt% reinforcement additive at 600 °C produced a notable 25.8% increase in maximum compression strength. A considerable improvement in microhardness was noticed, reaching 20.9% with a reinforcement addition of 2 wt%.
2. Sintering conditions had potential effects on improving the density and hardness values. For instance, the sample with 2 wt% AgNPs addition, sintered at 600 °C, recorded 34.70 VH1, while sample sintered at 550 °C recorded 29.52 VH1.
3. Ag nanoparticles’ sizes and dislocation distributions have a great effect on sample strength as they form barriers to dislocation movements that result in improved mechanical properties.
4. Increased nano-reinforcement content can lead to agglomeration, if not strictly controlled and addressed.
5. The formation of intermetallic bonding spots has a positive contribution to the strengthening mechanism of mechanical properties.

## Data Availability

The datasets used and/or analyzed during the current study available from the corresponding author on reasonable request.

## References

[1] Evans, A.* et al. Metal matrix composites*. (Springer, 2003).

[2] Clyne, T. W. & Hull, D. *An introduction to composite materials*. (Cambridge university press, 2019).

[3] Ahmad, Z. *Aluminium alloys: new trends in fabrication and applications*. (BoD–Books on Demand, 2012).

[4] Sharma VK, Kumar V (2019). Development of rare-earth oxides based hybrid AMCs reinforced with SiC/Al2O3: Mechanical and metallurgical characterization. J. Market. Res..

[5] Casati R, Vedani M (2014). Metal matrix composites reinforced by nano-particles—a review. Metals.

[6] Ramanathan A, Krishnan PK, Muraliraja R (2019). A review on the production of metal matrix composites through stir casting–Furnace design, properties, challenges, and research opportunities. J. Manuf. Process..

[7] Basavarajappa P, Parashivamurthy K (2017). Synthesis and tribological characterization of in-situ prepared Al-TiC composites. Am. J. Mater. Sci..

[8] Mohapatra S, Chaubey AK, Mishra DK, Singh SK (2016). Fabrication of Al–TiC composites by hot consolidation technique: Its microstructure and mechanical properties. J. Mater. Res. Technol..

[9] Carreño-Gallardo C, Mendoza-Duarte J, Lopez-Melendez C, Estrada-Guel I, Martinez-Sanchez R (2015). Evaluation of mechanical properties of aluminum alloy (Al-2024) reinforced with carbon-coated silver nanoparticles (AgCNP) metal matrix composites. Microsc. Microanal..

[10] Pitchayyapillai G, Seenikannan P, Balasundar P, Narayanasamy P (2017). Effect of nano-silver on microstructure, mechanical and tribological properties of cast 6061 aluminum alloy. Trans. Nonferrous Metals Soc. China.

[11] Arockiasamy A (2011). Sintering behaviour of Al-6061 powder produced by rapid solidification process. Powder Metall..

[12] Arshad H, Saleem M, Pasha U, Sadaf S (2022). Synthesis of Aloe vera-conjugated silver nanoparticles for use against multidrug-resistant microorganisms. Electron. J. Biotechnol..

[13] Morelhão, S. L. Computer simulation tools for X-ray analysis. *Graduate Texts in Physics *(Springer, Cham, 139, 2016).

[14] Alam MT, Ansari AH (2015). X-ray diffraction analysis and microstructural examination of al-sic composite fabricated by stir casting. Int. J. Sci. Technol. Manag.

[15] Rao JB, Kush D, Bhargava N (2012). Production and characterization of nano structured silicon carbide by high energy ball milling. J. Miner. Mater. Char. Eng..

[16] Asoro M, Damiano J, Ferreira P (2009). Size effects on the melting temperature of silver nanoparticles: In-situ TEM observations. Microsc. Microanal..

[17] Boyer, H. E. & Gall, T. L. Metals handbook; desk edition (1985).

[18] Swanson HE (1953). Standard X-ray diffraction powder patterns.

[19] Surega R, Anita B, Ramakrishnan S, Gunasekaran K, Nakkeran S (2020). Synthesis and characterization of AgNps using plant extracts. Int. J. Curr. Microbiol. Appl. Sci.

[20] Sendi R (2022). Grain size and sintering temperatures effects on the mechanical properties of ZnO nanoparticle-based varistor ceramics. J. Umm Al-Qura Univ. Appl. Sci..

[21] Rodriguez-Albelo LM (2023). Limits of powder metallurgy to fabricate porous Ti35Nb7Zr5Ta samples for cortical bone replacements. J. Mater. Res. Technol..

[22] Garner S, Ruiz E, Strong J, Zavaliangos A (2014). Mechanisms of crack formation in die compacted powders during unloading and ejection: An experimental and modeling comparison between standard straight and tapered dies. Powder Technol..

[23] Christy T, Murugan N, Kumar S (2010). A comparative study on the microstructures and mechanical properties of Al 6061 alloy and the MMC Al 6061/TiB2/12p. J. Miner. Mater. Char. Eng..

[24] Sharma R, Bisen D, Shukla U, Sharma B (2012). X-ray diffraction: A powerful method of characterizing nanomaterials. Recent Res. Sci. Technol..

[25] Alhajeri SN, Al-Fadhalah KJ, Almazrouee AI, Langdon TG (2016). Microstructure and microhardness of an Al-6061 metal matrix composite processed by high-pressure torsion. Mater. Char..

[26] Venkatesham M, Ayodhya D, Madhusudhan A, Veerabhadram G (2012). Synthesis of stable silver nanoparticles using gum acacia as reducing and stabilizing agent and study of its microbial properties: A novel green approach. Int. J. Green Nanotechnol..

[27] Nazaruddin A, Krishnakumar T (2015). Effect of addition of nanoparticles on the mechanical properties of aluminium. Int. J. Eng. Res..

[28] Kasraei S, Azarsina M (2012). Addition of silver nanoparticles reduces the wettability of methacrylate and silorane-based composites. Braz. Oral Res..

[29] Batool SA, Ahmad A, Wadood A, Mateen A, Hussain W (2018). Development of lightweight aluminum-titanium alloys for aerospace applications. Key Eng. Mater..

[30] Krishna MK, Karthik M (2014). Evaluation of hardness strength of aluminium alloy (AA6061) reinforced with silicon carbide. Int. J. Recent Technol. Mech. Electr. Eng. (IJRMEE).

[31] Dunstan MK, Paramore JD, Fang ZZ (2018). The effects of microstructure and porosity on the competing fatigue failure mechanisms in powder metallurgy Ti-6Al-4V. Int. J. Fat..

